# Preparatory studies of composite mesenchymal stem cell islets for application in intraportal islet transplantation

**DOI:** 10.3109/03009734.2010.524320

**Published:** 2011-02-11

**Authors:** Ida Rasmusson Duprez, Ulrika Johansson, Bo Nilsson, Olle Korsgren, Peetra U. Magnusson

**Affiliations:** Uppsala University, Department of Oncology, Radiology and Clinical Immunology, Division of Clinical Immunology, The Rudbeck Laboratory, UppsalaSweden

**Keywords:** Human islets of Langerhans, human mesenchymal stem cells, human multipotent stromal cells

## Abstract

**Background:**

Low engraftment and adverse immune reactions hamper the success rate of clinical islet transplantation. In this study, we investigated the capacity of human mesenchymal stem cells (MSCs) to adhere to human islets of Langerhans and their effects in immune modulation and during blood interactions *in vitro.*

**Methods:**

Composite MSC–islets were formed by suspension co-culture, and the phenotype was evaluated by confocal microscopy. Islet function was assessed by dynamic insulin release in response to glucose *in vitro.* Mixed lymphocyte–islet reactions (MLIR) and the tubing blood loop model were utilized as *in vitro* tools to analyse the effect of MSCs on the innate and adaptive immune reactions triggered by the islets.

**Results:**

MSCs rapidly adhered to islets and spread out to cover the islet surface. Insulin expression and secretion were sustained with the MSC coating. MSC-coated islets showed unaffected reactions with blood *in vitro* in comparison to control islets. Furthermore, MSCs suppressed lymphocyte proliferation induced by islet cells in MLIR.

**Conclusion:**

We conclude that it is possible to create composite MSC–islets to enable delivery of the MSCs by utilizing the adhesive capacity of the MSCs. This could have beneficial immunosuppressive effects in optimizing pancreatic islet transplantation.

## Introduction

Multipotent mesenchymal stromal/stem cells (MSCs) are adherent cells that reside in the stroma, where they produce stromal components (1,2). Through their multipotency MSCs can differentiate towards fat, bone, or cartilage upon the correct stimuli (1). MSCs have been shown to possess an immunosuppressive capacity and to protect against activated immune responses (3–5). MSCs can also suppress adaptive immune reactions (6–9) and have shown a dramatic positive effect on severe graft-versus-host disease in humans (10,11). The beneficial effects of MSCs make them attractive as tolerance-inducing cells during cell transplantation (3,12,13). Transplantation of islets containing insulin-producing beta cells is currently evaluated as a treatment for patients with life-threatening hypoglycaemic episodes and unsatisfactory glycaemic control despite careful insulin therapy (14). One key factor for successful islet transplantation is to use a high number of islets, in part due to loss of islets in response to adverse immune reactions (15–17). The immunosuppressive capacity of MSCs, alongside the ability to improve regeneration of damaged tissue (18–21) and to provide a supportive stromal environment, could benefit islet transplantation. MSCs have been isolated from pancreatic ductal epithelium and may therefore have a natural role to protect and compose a suitable environment for the endocrine cells in the native pancreas (22). At the same time, the islets provide a surface for adherence of MSCs which could improve the survival of MSCs. In this study, we have analysed the interactions between islets and MSCs concerning the potential of MSCs to attach to the islets, and the consequences this has on insulin secretion of the islets, blood contact, and immune modulation by the MSCs *in vitro*.

Delivery of islets and MSCs separately during intraportal transplantation will lead to deposition of MSCs to other sites than the islets. The possibilities to bind the MSCs to the surface of the islets will not only make it possible to target the MSCs to the same site as the islets in the clinical intraportal transplantation, but also increase the likelihood of initial contact between the MSCs and the islets. Here we show that the blood interactions of the MSC–islets in the tubing blood loop model have no further negative effect upon the coagulation or the complement system.

## Material and methods

### Isolation of human islets of Langerhans

Human islets of Langerhans were isolated by the isolation laboratory of the Nordic Network in Uppsala, Sweden, as previously described (23), using a modified semi-automated digestion-filtration method (24–26). Pancreases were obtained from brain-dead donors after appropriate consent for multi-organ donation. Islet preparations with a purity ranging from 70% to 80% have been analysed in this study. Purity was estimated by dithizone staining of endocrine islet tissue. Islets were cultured in islet medium: Connaught Medical Research Laboratories (CMRL) 1066 culture medium with 10% human ABO serum, 2.4 μM L-glutamine, 12 μM Hepes, 12 μM nicotinamide, 6 μM sodium pyruvate, 50 μg/mL gentamycine, 20 μg/mL ciproxine, and 0.25 μg/mL fungizone.

### Isolation and expansion of adult human MSCs

The human MSCs used in this study were generously provided by Professor Katarina Le Blanc, Karolinska Institutet, Stockholm, Sweden. The MSCs were isolated and expanded from bone marrow (BM) as previously described (27) following the approval by the ethics committee at Huddinge University Hospital. The cells were cultured in MSC medium consisting of Dulbecco's modified Eagle's medium-low glucose (DMEM-LG), supplemented with 10% heat-inactivated foetal bovine serum (FBS) (from PAA, Pasching, Austria) and 1% antibiotic solution. The cells were classified as MSCs based on plastic adherence, differentiation to bone and fat, and expression of surface markers (28). MSCs in passages 3–9 from 13 donors were used in this study.

### Adherence and spreading of MSCs on human islets

Approximately 1,000 human islets were mixed with 1 × 10^5^, 2.5 × 10^5^, or 5 × 10^5^ MSCs in 1 mL of islet culture medium. To evaluate the degree of coating, the MSCs were stained with CellTracker Green according to the manufacturer's instructions (Molecular Probes, Eugene, OR, USA). The MSCs and islets were incubated at 37°C for 3 hours in cell suspension tubes, mixed gently every 30 minutes, and subsequently cultured in islet medium (*n* = 13). Evaluation of the adherence and spreading of the MSCs on the islet surface was performed by confocal laser scanning microscopy (Zeiss LSM 510 Meta, Carl Zeiss AG, Göttingen, Germany). To analyse the distribution of MSCs, within the islets, islet preparations were fixed for 1 hour at room temperature (RT) in 4% formaldehyde, washed in Phosphate Buffered Saline (PBS) overnight, incubated in 20% sucrose for 12–24 hours, before being embedded in Optimal Cutting Temperature (OCT) medium (Tissue Tech, Sakura Finetechnical, Tokyo, Japan) and snap-frozen in liquid nitrogen. Sections of 10 mm were counterstained with 4′,6′-diamidino-2-phenylindole (DAPI) (10 mg/mL, Fluka, Sigma-Aldrich, St.Louis, MO, USA).

### Measurement of insulin release and insulin DNA

The functionality of the islets coated with MSCs compared with control islets from the same isolation was tested after 48 hours. Twenty hand-picked MSC-coated and non-coated control islets were challenged in a dynamic perifusion system, beginning with a low-glucose concentration (1.67 mmol/L), followed by a high-glucose challenge (20 mmol/L) and then re-exposure to the low-glucose concentration. Fractions were collected at 6-minute intervals over 126 minutes and analysed for insulin content using commercial Enzyme-linked immunosorbent assay (ELISA) kits (Mercodia, Uppsala, Sweden). Stimulation index was calculated: (average of high-glucose samples)/(average of low-glucose samples). For measurements of insulin/DNA ratio, 20 hand-picked MSC-coated and control islets were collected and analyzed for insulin content by ELISA (Mercodia). Quant-iT picogreen dsDNA assay kit (Invitrogen, Carlsbad, CA, USA) was used for DNA measurements and analysed by a fluoroscan ascent Fluorometer Luminometer (FL) (Thermo Electron Corp., Waltham, MA, USA).

### Immunohistochemistry

Peripheral blood mononuclear cells (PBMC) were isolated from healthy human volunteers. The blood was diluted in PBS, and PBMC were separated on a Ficoll gradient (1.077 g/L; Axis-Shield PoC AS, Oslo, Norway). After three washes in PBS, the cells were resuspended in Roswell Park Memorial Institute (RPMI) 1640 medium supplemented with 10% human pooled AB serum, 2 mM L-glutamine, 100 U/mL penicillin, and 100 μg/mL streptomycin (complete RPMI medium). To obtain single islet cells the islets were washed twice in PBS and incubated in Accutase (Sigma-Aldrich) for 10 minutes at 37°C. The reaction was stopped by addition of an equal volume of cold FBS, and the cell suspension was carefully vortexed to dissociate the islets. Responder PBMC (100,000) and stimulator pancreatic single cells (100,000) were mixed in 200 μL of complete RPMI medium in triplicates, without or with 10,000 (10%) or 1,000 (1%) of MSCs. Stimulator cells and MSCs were irradiated and co-cultured for 5 days. The proliferation of responder PBMC was measured after another 24 hours of tritium-labelled thymidine incorporation (1 μCi, Radiochemical Centre, Amersham, UK). The cells were harvested automatically on glass fibre filters, using a Tomtec harvesting machine (Harvester 96, Tomtec, Orange, CT, USA). Two independent experiments with two islet preparations and in total four different MSCs were tested (*n* = 6).

### Transplantation of composite islets

Approximately 200 control human islets or an equal number of MSC-coated human islets were transplanted under the kidney capsule of C57BL/6/sca nu/nu mice (Scanbur AB, Sollentuna, Sweden). After 2 weeks, the kidneys were removed and stained for insulin (*n* = 3).

To perform transplantation of syngeneic composite grafts, mouse islets were isolated and hand-picked from C57BL/6/sca (Scanbur, Sweden), and approximately 200 islets were coated with 125,000 murine MSCs derived from C57BL/6/sca, a kind gift from Professor Rafi Gorodetsky, Sharett Institute of Oncology, Hadassah-Hebrew University Medical Center, Jerusalem, Israel (29). The islets and MSC–islets were transplanted under the kidney capsule, and grafts were retrieved 28 days after transplantation (*n* = 3). Animal handling was performed with ethical permission approved by the authorities and according to the UKCCCR guidelines for the welfare of animals in experimental neoplasia (30).

### One-way mixed lymphocyte–islet reactions (MLIR)

Insulin expression in transplanted human islets was analysed in paraffin-embedded tissue. Kidney biopsies were fixed in 4% paraformaldehyde for 24 hours and prepared for paraffin embedding. Sections were stained for insulin (guinea-pig anti-insulin, Dako, Glostrup, Denmark) following a secondary antibody (goat anti-rabbit Envision, Dako). Finally, the sections were developed in AEC (3-amino, 9-ethyl-carbazole, Dako) and counterstained with haematoxylin. Transplanted syngeneic MSC–islets and control islets were prepared as described above including a decalcification step according to manufacturer's instructions (Pargeny, Histolab, Gothenburg, Sweden) of the MSC–islet grafts before sectioning and thereafter haematoxylin and eosin staining.

### The tubing blood loop model

Untreated control human islets and MSC–islets were exposed to fresh human ABO-compatible blood using a loop system consisting of polyvinyl chloride (PVC) tubings with a heparinized inner surface (31). The loop system was placed on a rocking apparatus in an incubator at 37°C to simulate a blood-flow inside the PVC tubing. Loops with 7 mL of human blood were prepared, and 100 μL of PBS (negative control) or 500–800 coated or uncoated islets in 100 μL of PBS were added (*n* = 5). The loops were then closed with a polypropylene connector and placed in the 37°C incubator on the rocking apparatus. One mL of blood was collected at 3 different time points (5, 10 and 30 min) in tubes containing 50 μL 0.2 M NaEDTA, pH 7.4, to determine platelet consumption, and plasma was prepared from the remaining blood in the sample for ELISA analysis of C3a and thrombin anti-thrombin (TAT) (Mercodia, Uppsala, Sweden).

### Statistical analysis

Statistics were calculated using the Statistica software (Statsoft Scandinavia AB, Uppsala Sweden). Results were compared using the non-parametric Wilcoxon test, and *P* values ≤ 0.05 were considered statistically significant.

## Results

### Adherence and spreading of MSCs

**Figure 1.**
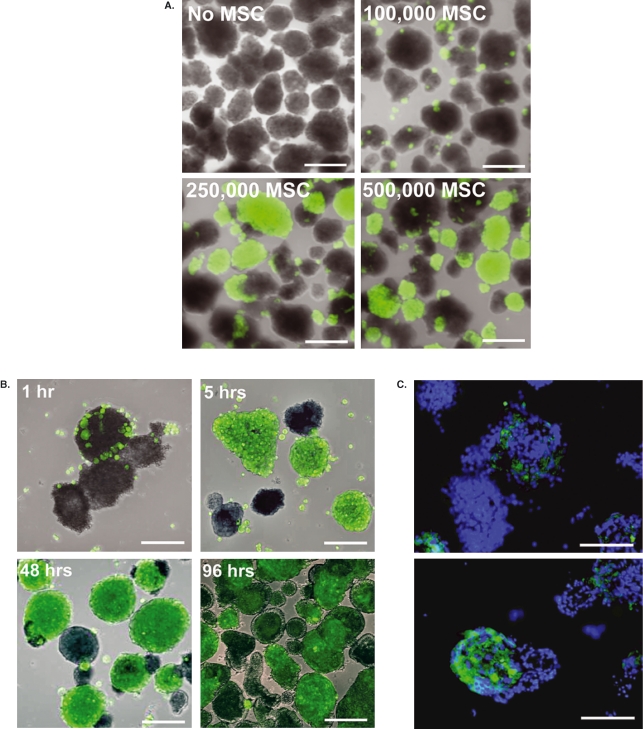
Coating of pancreatic islets with MSCs. Human islets were coated with CellTracked MSCs (green). A: Dose-dependent coating of islets with 100,000, 250,000, or 500,000 MSCs after 48 hours of culture. B: The initial adherence and the following spreading of MSCs after 1, 5, 48, and 96 hours of culture. One representative experiment is shown. C: MSCs within the islet core after 48 hours of co-culture, nuclei in blue. Scale bars = 100 μm.

MSCs showed a dose-dependent coating of the islets. With addition of 100,000 MSCs to approximately 1,000 islets, only a sporadic binding to the islet surface was detected. Increased coating was observed when 250,000 and 500,000 MSCs were used (Figure 1A). A further increase in the number of MSCs did not result in increased coverage. Rather there were many single cells in suspension, whereas a proportion of the islets remained free of coating. When pancreatic islets and MSCs were incubated together, the MSCs adhered to the islets within 1 hour (Figure 1B). After 5 hours, the MSCs had initiated spreading and covered most of the islet surface. After 24–48 hours, the adherence of the cells was completed with no apparent improvement of coating nor loss of MSC attachment for up to 96 hours, which was the selected end-point of the coating experiments (Figure 1B). In addition to coating the surface of the islets, MSCs were also found within the islet core (Figure 1C).

### Insulin secretion in composite MSC–islets

**Figure 2.**
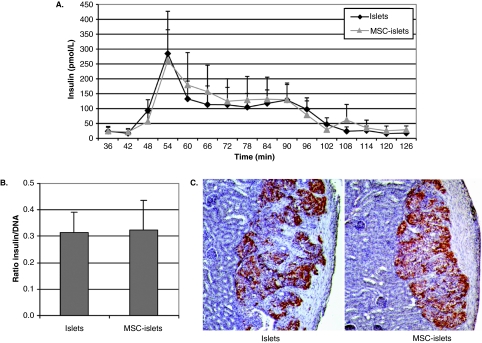
Insulin secretion and expression in MSC-coated islets. A: Insulin secretion was measured in a dynamic perifusion system (*n* = 5) where control islets (black diamonds) or composite MSC–islets (light triangles) were initially challenged with low-glucose (1.67 mmol/L) followed by high-glucose stimulation (20 mmol/L, 42–78 min). B: The ratio of insulin versus total DNA (pmol × L^−1^ × ng^−1^) in islet homogenates (*n* = 3). C: Insulin-containing cells survived *in vivo*, indicated by insulin-staining of grafts of human islets and MSC–islets transplanted under the kidney capsule of nude mice retrieved after 2 weeks (*n* = 3).

Functionality of the islets was investigated by insulin secretion in a dynamic perifusion system, where the islets were stimulated with different glucose concentrations. The shift from low to high glucose solution stimulates a rapid release of insulin. On average, the coating of islets with MSCs did not affect the insulin secretion by the islets (Figure 2A, *n* = 5). The stimulation index was not significantly different between control islets and composite MSC–islets (11.0 ± 5.2 for control islets and 9.5 ± 3.6 for MSC–islets, average ± SEM, *n* = 5). Additionally, insulin/DNA ratio (pmol × L^−1^ × ng^−1^) showed no difference between control islets and composite MSC–islets, even though the MSCs contributed with DNA in the insulin and DNA ratio of MSC–islets (Figure 2B, *n* = 3). Human islets or composite MSC–islets were transplanted under the kidney capsule of nu/nu mice, and all grafts were positively stained for insulin when retrieved after 2 weeks (Figure 2C, *n* = 3).

### Syngeneic transplantation of mouse MSC–islets

**Figure 3.**
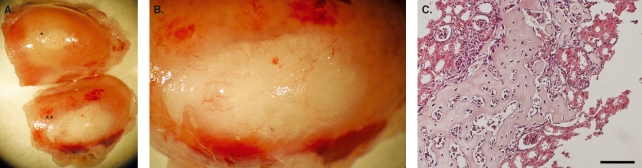
Syngeneic transplantation of mouse MSC–islets. A: Control mouse islets (*) and mouse MSC–islets (**) were transplanted under the kidney capsule of mice and retrieved after 28 days *in vivo*, showing enlargement of the MSC–islet graft (**) in comparison to control islet graft (*) (*n* = 3). B: Close up of the MSC–islet graft. C: Section of mouse MSC–islet grafts after decalcification showing bone formation of differentiated MSCs. Bar = 100 μm.

Transplantation of mouse islets, control islets, or MSC–islets was performed under the kidney capsule of mice (*n* = 3). The grafts were removed 28 days after transplantation. By then, the MSC–islet grafts were differentiated into bone, and the graft was enlarged in comparison to control (Figure 3A, B). The bone formation was further verified after sectioning followed by haematoxylin and eosin staining (Figure 3C).

### MSCs and islet cells in mixed lymphocyte–islet reactions (MLIR)

**Figure 4.**
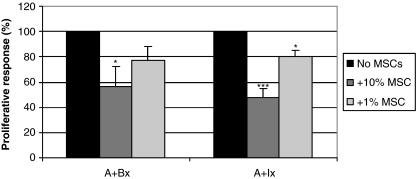
MSCs suppressed lymphocyte proliferation against dissociated islets in MLIR. In one-way proliferative assays between PBMCs and pancreatic islet single cells (A+Ix), the addition of 10% (dark grey bars) and 1% MSCs (light grey bars) significantly suppressed proliferation, compared to the positive control without MSCs (black bars). The relative inhibition was similar to the control where the lymphocytes reacted against allogeneic irradiated lymphocytes (A+Bx). The figure shows an average of six different islets/PBMC/MSC donor combinations (***P < 0.001; *P < 0.05).

To analyse the immunological effects of MSCs on immune reactions induced by islets, we cultured peripheral blood mononuclear cells (PBMC) with dissociated allogeneic islet cells with or without allogeneic MSCs in suspension culture during 6 days and studied lymphocyte proliferation by incorporation of ^3^H-thymidine during the last 24 hours. MSCs and stimulator cells were irradiated not to proliferate, to yield a one-way MLIR. The addition of 10% or 1% MSCs significantly suppressed proliferation (Figure 4). The positive control in which the effector cells reacted against allogeneic PBMC induced a proliferation of 42,493 ± 15,317 counts per minute (cpm) and reaction against islet cells 9,721 ± 3,796 cpm. The results were adjusted to per cent response relative to the respective control without MSCs to adjust for donor differences.

### MSC–islets in the tubing blood loop model

**Figure 5.**
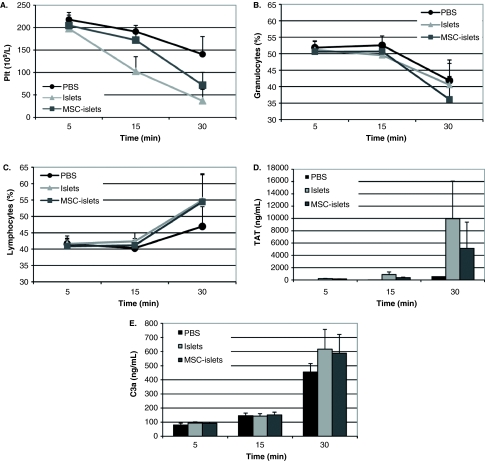
Blood interactions *in vitro* of control islets and MSC–islets. Control islets (light grey) and human MSC–islets (dark grey) were exposed to human ABO-compatible blood in the tubing blood loop model, and PBS (black) was used as control. Platelet counts (A) and granulocytes (B) decreased, whereas lymphocytes (C) increased. There was no statistically significant difference between control islets and composite grafts. Activation of coagulation was investigated by measurements of thrombin anti-thrombin (TAT) levels, which increased after 30 minutes of incubation in both control islets and MSC–islets compared to PBS (D). Complement activation was triggered in all three groups, shown by increased levels of C3a (E).

*In vitro* studies of islets and MSC–islets interactions with ABO-compatible blood in tubing loops coated with heparin were investigated. The results showed a similar reduction in platelets (Figure 5A) and granulocytes (Figure 5B) and increase in lymphocytes (Figure 5C) during 30 minutes of incubation in both groups due to infiltration of cells in blood clots. A similar activation of coagulation occurred by control islets and MSC–islets as shown by increased levels of thrombin anti-thrombin (TAT) (Figure 5D). This effect was also shown by activation of the complement by increased levels of C3a (Figure 5E).

## Discussion

We have evaluated the possibility to coat human islets of Langerhans with human MSCs and investigated their role in islet function in different *in vitro* models symbolizing the scenario of clinical islet transplantation.

Intravenous injections of MSCs in rodents show deposit of the injected cells in the lungs (32), indicating that intravenous administration of the cells results in entrapment of the cells. The distribution of MSCs depends on the site of infusion (33–35), and injuries can alter the distribution pattern with an increased cell migration to the site of injury (35,36). We incubated MSCs with islets and studied adhesion and spreading to the islets for up to 96 hours. In addition to the good capacity to coat islets, MSCs were also found within the islet core. Coating of islets with MSCs did not adversely affect the islet function as confirmed by measuring the insulin release elicited by MSC–islets after 48 hours of culture. Our results show that the interactions between islets and MSCs are rapid with a high degree of binding within the first 24 hours. This is compared to coating with aortic endothelial cells (EC) that required up to 7 days for an optimal coverage of the islet surface (23). The addition of MSCs to composite EC–islets has previously shown enhanced adhesion of the microvascular EC to the islet surface. Besides facilitating binding of EC to the islet surface, MSC-conditioned medium stimulated proliferation of EC (37). Positive effects of MSCs on revascularization in pancreatic islets have also been shown *in vivo* (38).

MSCs can suppress various immune responses (reviewed in (39)). In this study, we have analysed *in vitro* the potential cellular rejection response that can be triggered by transplanted islets in MLIR. MSCs significantly reduced proliferation of lymphocytes, with possible importance in co-transplantation of islets with MSCs, by inducing a local immunosuppressive milieu surrounding the islets. We dissociated the islets to optimize the contact between lymphocytes and islet cells, since whole islets were unable to stimulate measurable levels of proliferation (data not shown). During the dissociation of islets, naturally other islet cells than beta cells, such as alpha, delta, and pancreatic polypeptide (PP) cells, but also endothelial cells, are released, and they most likely contribute to stimulate proliferation. Beta cells express low levels of human leukocyte antigen (HLA) class I and do not express HLA II (40). Only a small fraction of the non-endocrine cells express HLA class DR, mainly macrophages and endothelial cells (41), which may explain the lower proliferation by responder lymphocytes. Other factors that can alter the immunogenicity of islets are the production of inflammatory cytokines (42) and the presence of acinar cells in the dissociated islets that can suppress the proliferative response of lymphocytes (43). The effect of composite MSC–islets on interactions with blood did not show any increased activation, which is beneficial for intraportal transplantation of composite grafts.

MSCs could play an important role in suppressing cellular rejection of transplanted islets. Fiorina et al. suggested that murine MSCs protect the pancreas from autoimmune destruction in a NOD mouse model by recruiting the MSCs to pancreatic lymph nodes to suppress T cells through the co-stimulatory molecule programmed death-ligand 1 (PD-L1) (44). Itakura and colleagues reduced rejection of transplantation of pancreatic islets by co-transplantation of MSCs. Diabetic rats receiving an intraportal co-infusion of MSCs, bone-marrow cells, and islets rejected the islets initially. However, half of them developed stable mixed chimerism and donor-specific immune tolerance, as shown by the engraftment of donor skin and second-set islet transplants and acute rejection of a third-party skin (45).

Undesired differentiation of cells should always be considered in transplantation studies using stem cells. We have chosen to use human MSCs and human islets of Langerhans. Our attempts to use syngeneic models with mouse MSCs in composite islets resulted in bone formation under the kidney capsule. This phenomenon was first shown by Friedenstein et al. in 1966 (46). Human MSCs appear to be less inclined to form bone *in vivo* unless the correct stimuli are present (47). In this study we have not experienced bone differentiation of human MSCs transplanted to nu/nu mice.

In this study, we wanted to determine the outcome of coating pancreatic islets with MSCs by investigating islet function and the effect on innate and adaptive immunity. To conclude, we show that MSCs can both cover the surface and colonize the inner core of pancreatic islets. The MSCs inhibited lymphocyte proliferation induced by islet cells, whereas the innate immune reactions were unaltered. Formation of composite islet grafts also facilitated delivery of MSCs together with the islets when transplanted under the kidney capsule. From these data we have shown that the MSCs can be bound to the surface of the islets to ensure delivery at the site of transplantation where they may have promising effects on immune regulation to support engraftment of transplanted islets of Langerhans.
